# Pretreatment with G-CSF Could Enhance the Antifibrotic Effect of BM-MSCs on Pulmonary Fibrosis

**DOI:** 10.1155/2019/1726743

**Published:** 2019-01-03

**Authors:** Feiyan Zhao, Wei Liu, Shaojie Yue, Lei Yang, Qingzhong Hua, Yan Zhou, Haipeng Cheng, Ziqiang Luo, Siyuan Tang

**Affiliations:** ^1^Department of Physiology, Xiangya School of Medicine, Central South University, Changsha, Hunan, China; ^2^College of Veterinary Medicine, Hunan Agricultural University, Changsha, Hunan, China; ^3^Xiangya Nursing School, Central South University, Changsha, Hunan, China; ^4^Department of Pediatrics, Xiangya Hospital, Central South University, Changsha, Hunan, China

## Abstract

Granulocyte colony-stimulating factor (G-CSF) can promote the repair of a variety of damaged tissues, but the underlying mechanisms have not yet been fully elucidated. Bone marrow mesenchymal stem cells (BM-MSCs) play an important role in the repair of damaged tissue. The aim of this study was to explore whether pretreating BM-MSCs with G-CSF can promote their ability of homing to the lung after in vitro transplantation via upregulating the CXCR4 expression, potentially markedly increasing the antifibrotic effect of BM-MSCs. The BM-MSCs pretreated with G-CSF were transplanted into a mouse on day 14 after bleomycin injection. The antifibrotic effects of BM-MSCs in mice were tested on day 21 by using pathological examination and collagen content assay. Pretreatment of BM-MSCs with G-CSF significantly promoted their ability of homing to the lung and enhanced their antifibrotic effects. However, knocking down the CXCR4 expression in BM-MSCs significantly inhibited the ability of G-CSF to promote the migration and homing of BM-MSCs to the lung and the resulting antifibrotic effects. We also found that G-CSF significantly increased the CXCR4 expression and AKT phosphorylation in BM-MSCs, and the AKT pathway inhibitor LY294002 significantly diminished the ability of G-CSF to upregulate the CXCR4 expression in BM-MSCs. Pretreatment of BM-MSCs with G-CSF promotes the homing of BM-MSCs to the lung via upregulating the CXCR4 expression, leading to a marked increase in the antifibrotic effects of BM-MSCs. This study provides new avenues for the application of BM-MSCs in the repair of different tissues.

## 1. Introduction

Idiopathic pulmonary fibrosis (IPF) is a disease characterized by diffuse interstitial inflammation and fibrosis, which is typified by fibroblast proliferation and massive extracellular matrix deposition [1]. The median survival of 2–5 years and the incidence have been increasing in recent years, but there is a lack of specific and effective therapeutics [2]. Therefore, it is important to search for novel treatments.

With the progress in regenerative medicine and tissue engineering, cell-based therapies are currently under investigation. Recent studies have suggested that bone marrow (BM) cells may be a reservoir of stem cells useful for tissue regeneration [3, 4]. BM-derived stem cells consist mainly of haematopoietic stem cells (HSCs), bone marrow mesenchymal stem cells (BM-MSCs), and endothelial progenitor cells (EPCs). MSCs are currently the most popular cell type for tissue-engineered stem cell applications. Studies have shown that MSCs can promote tissue repair via paracrine signals or by directly differentiating into substitute functional cells of the damaged tissue [5]. The clinical application of MSCs has made significant breakthroughs in the treatment of various diseases, including respiratory diseases, cardiovascular diseases, cirrhosis, and neurological diseases [6–9].

G-CSF shows a protective effect on many diseases. G-CSF can mobilize HSCs to migrate to damaged parts of the heart and induce myocardial regeneration [10, 11]. It can also mobilize BM-MSCs to infiltrate into the brain, replenish the neural lineage cells, and contribute to neurogenesis in the brains of mice with Alzheimer's disease [12]. It was also found that G-CSF can significantly reduce liver tissue damage through promoting hepatocyte regeneration [13]. However, as far as the lung injury is concerned, Zhang et al. found that systemic administration of G-CSF significantly inhibited bleomycin-induced lung fibrosis in mice, but Adachi et al. indicated that systemic administration of G-CSF significantly aggravated bleomycin-induced pulmonary fibrosis [14, 15]. Therefore, there is still great controversy over the systemic treatment of lung injury with G-CSF.

Adopting appropriate pretreatment strategies can provide a simple and effective method to promote the repair ability of MSCs. Recent studies showed that short-term exposure of MSCs to hypoxia could downregulate apoptosis-related signalling pathways in MSCs and increase cell survival [16, 17]. Hypoxia pretreatment prior to transplantation can also enhance the repair ability of MSCs [18, 19]. In this study, we used G-CSF to pretreat BM-MSCs and observed the effect of G-CSF-pretreated BM-MSCs on bleomycin-induced pulmonary fibrosis.

For the first time, this study confirmed that G-CSF promotes the homing of BMSCs to damaged lung tissue by upregulating the CXCR4 expression, thereby enhancing the antifibrotic effects of BM-MSCs. Our research may provide new ideas for the clinical application of G-CSF and MSCs.

## 2. Materials and Methods

### 2.1. Animal Model and Experimental Design

Female C57BL/6 mice were obtained from Jingda Laboratory Animal Company (Changsha, China). After being anaesthetized with pentobarbital sodium, the mice received an intratracheal injection of 50 *μ*L of bleomycin (BLM) (3.5 mg/kg) (Nippon Kayaku, Japan) on day 0. To study the antifibrotic effects of BM-MSCs pretreated with rmG-CSF (PeproTech, USA), C57BL/6 mice were randomly assigned to one of the following groups: (1) control group, intratracheal saline plus tail vein injection of phosphate-buffered solution (PBS); (2) BLM group, intratracheal BLM plus tail vein injection of PBS; (3) BLM + rmG-CSF- (30 ng/mL) pretreated BM-MSC (1 × 10^5^ in 100 *μ*L) group, intratracheal BLM plus rmG-CSF-pretreated BM-MSC infusion into the tail vein; (4) BLM + rmG-CSF- (30 ng/mL) pretreated BM-MSC (3 × 10^5^ in 100 *μ*L) group; (5) BLM + rmG-CSF- (30 ng/mL) pretreated BM-MSC (1 × 10^6^ in 100 *μ*L) group; (6) BLM + BM-MSC (1 × 10^5^ in 100 *μ*L) group, intratracheal BLM plus BM-MSC infusion into the tail vein; (7) BLM + BM-MSC (3 × 10^5^ in 100 *μ*L) group; and (8) BLM + BM-MSC (1 × 10^6^ in 100 *μ*L) group. rmG-CSF-pretreated BM-MSCs and nontreated BM-MSCs were infused into the tail vein on day 14 after BLM injection, and lung tissues were harvested on day 21.

### 2.2. Histopathology

Lung tissues were fixed with 4% paraformaldehyde solution and then embedded in paraffin for the preparation of tissue sections for pathological examination. The sections were examined after being stained with haematoxylin and eosin (H&E) or Masson's trichrome.

### 2.3. Hydroxyproline Assay

The collagen content was examined using hydroxyproline (HYP) kits (Njjcbio, China) according to the manufacturer's instructions.

### 2.4. Real-Time PCR

Total RNA was extracted from lung tissues and BM-MSCs using RNAiso Plus (TaKaRa, Japan), and cDNA synthesis was performed using a First-Strand cDNA Synthesis Kit. SYBR Green signals were detected using a Bio-Rad CFX96 real-time PCR detection system. The real-time PCR primer sequences are shown in Table 1.

### 2.5. Western Blot

Tissue homogenates were centrifuged at 12,000 rpm for 30 minutes at 4°C. Thirty micrograms of protein was separated by 10% SDS-PAGE and electroblotted onto polyvinylidene difluoride (PVDF) membranes, which were blocked with 5% TBST-milk at room temperature for 1 hour, incubated at 4°C overnight with primary antibody (*α*-SMA, rabbit polyclonal antibody, Abcam, UK; pro-SPC, rabbit polyclonal antibody, Abcam, UK; AKT, rabbit polyclonal antibody, CST, USA; p-AKT, rabbit polyclonal antibody, CST, USA; and *β*-actin, mouse monoclonal antibody, CMCTAG, USA) in 1% TBST-milk, and incubated for 1 hour with HRP-conjugated secondary antibody (1 : 5000 dilution, goat anti-rabbit IgG antibody, Boster, China; 1 : 3000 dilution, goat anti-mouse IgG antibody, Millipore, USA). Protein bands were subsequently detected using enhanced chemiluminescent (ECL) reagents (Millipore, USA).

### 2.6. BM-MSC Isolation and Culture

BM-MSCs were extracted from the femurs of 4-week-old C57BL/6 mice, and GFP-labelled BM-MSCs were purchased from Cyagen Biosciences Incorporation (Guangzhou, China). These cells were cultured in DMEM/F12 (Gibco, USA) supplemented with 10% foetal bovine serum (FBS) (Gibco, USA). In the details of BM-MSC isolation and purification, the BM was flushed with DMEM/F12 medium, and the isolated cells were cultured in 5% CO_2_ at 37°C. To improve the BM-MSCs purification rate, the cells were washed, and the culture medium was changed after 48 hours. Subsequently, the medium was changed every 2–3 days, and the cells were maintained until they reached approximately 80% confluence. Cells were then trypsinized, replated, and cultured for isolation and purification. BM-MSCs were identified by flow cytometric analysis with antibodies against CD29, CD31, CD34, CD44, and Sca-1. The stemness of BM-MSCs was confirmed by inducing differentiation into adipocytes and osteocytes (data not shown).

### 2.7. Single-Cell Suspension of Lung Tissue Was Prepared

The mice were anesthetized and the pulmonary circulation was perfused with saline to flush the cells in the blood. Lung tissue was removed and mechanically minced. The minced lung tissue was digested with RPMI 1640 complete medium containing 0.2% *w*/*v* collagenase I at 37°C for about 3 hours, and digestion was terminated by the ice bath. A 200-mesh stainless steel mesh was used to filter the cells, and the filtrate was collected by centrifugation at 1500 rpm for 5 minutes. The supernatant was discarded and the cells were rinsed twice with PBS. After the red blood cells were removed, the collected samples were pooled and resuspended. The cell density was then adjusted to 2 × 10^6^ cells/mL.

### 2.8. Flow Cytometry

BM-MSCs were stained with anti-CXCR4 antibody, and flow cytometry (FCM) was used to detect CXCR4 expression on cell surface. Antibodies of the corresponding isotype served as the isotype control.

### 2.9. Cell Proliferation Assay

For various treatment conditions, the BM-MSCs were plated in 96-well plates and cultured for 24 hours. BM-MSC proliferation was investigated using a Cell Counting Kit 8 (Beyotime, China) according to the manufacturer's instructions.

### 2.10. Transfection of BM-MSCs with LV-CXCR4-RNAi

Well-conditioned BM-MSCs were prepared in complete culture medium, and a suspension of 3 × 10^4^ cells/mL was incubated into a 96-well plate at 100 *μ*L per well. BM-MSCs were transfected with negative control RNAi (MOI = 10, 20, or 30; lentiviral vector titer = 1 × 10^9^; sequence, TTCTCCGAACGTGTCACGT). The fluorescence intensity of the cells 72 hours after transfection was observed by fluorescence microscopy, and the optimal MOI and transfection conditions were determined. Under optimal conditions (MOI = 30), BM-MSCs transfected with LV-CON-RNAi, LV-CXCR4-RNAi(01) (sequence, AGATCCTTTCCAAAGGAAA), LV-CXCR4-RNAi(02) (sequence, GTTTCAATTCCAGCATATA), or LV-CXCR4-RNAi (03) (sequence, TGACTATACCTGACTTCAT) were collected.

### 2.11. BM-MSC Homing Assay

Before the homing assay, GFP-labelled BM-MSCs were pretreated with or without rmG-CSF (30 ng/mL) for 24 hours. Then, the nontreated or rmG-CSF-pretreated BM-MSCs (1 × 10^5^, 3 × 10^5^, or 1 × 10^6^ in 100 *μ*L) were infused into the tail vein of the control or BLM-treated mice on day 14 after BLM injection. Mice were sacrificed on day 21 and the lungs were harvested. The number of GFP-labelled cells was quantified by FCM.

### 2.12. Transwell Assay

Before the transwell assay, BM-MSCs were pretreated with or without rmG-CSF (30 ng/mL) for 24 hours. Then, we seeded the nontreated BM-MSCs or rmG-CSF-pretreated BM-MSCs into the upper transwell compartments in 24-well plates. The normal or BLM-induced lung tissue on day 14 after BLM administration was harvested, cut into pieces (1 mm^3^), and incubated into the lower chamber. After allowing the BM-MSCs to migrate for 18 hours, the transwell membranes were washed, and the nonmigrating BM-MSCs on the upper side of the membrane were removed with a swab. The migrated BM-MSCs were imaged and counted after crystal violet staining.

### 2.13. Statistical Analysis

For multiple groups, the data were analyzed using one-way ANOVA followed by post hoc test to compare differences between the groups. For two groups, the data were analyzed using Student's *t*-test. Differences between the groups were considered significant at *P* < 0.05. All analyses were performed using GraphPad Prism 6.0. Data are shown as mean ± SD.

## 3. Results

### 3.1. Pretreatment with G-CSF Enhances the Ability of BM-MSCs to Inhibit BLM-Induced Pulmonary Fibrosis

**Figure 1:**
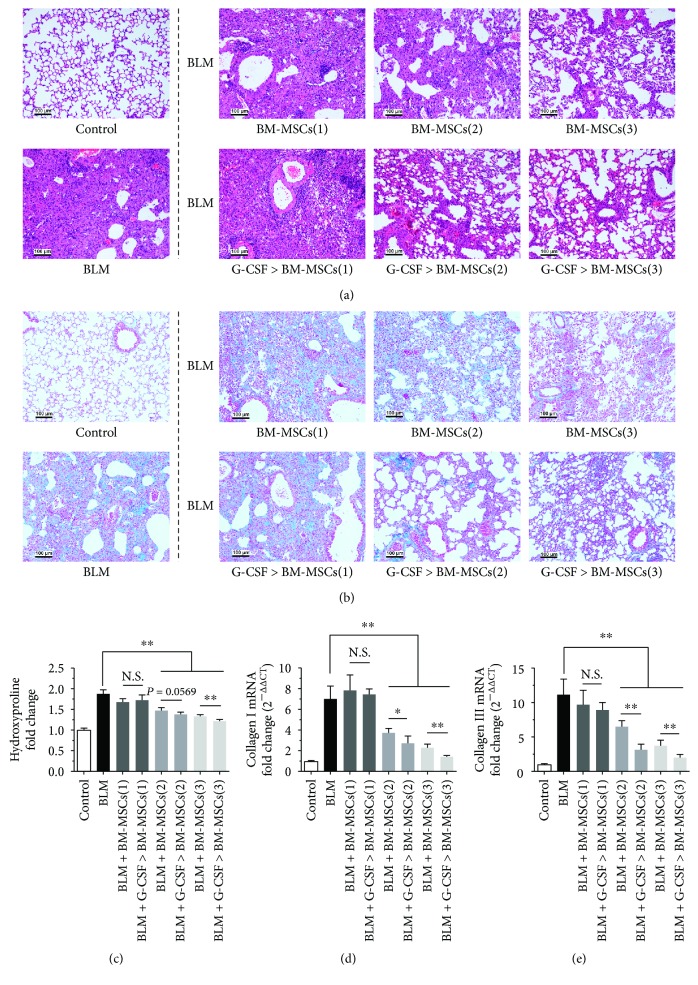
Pretreatment with G-CSF enhances the ability of BM-MSCs to inhibit BLM-induced pulmonary fibrosis. C57BL/6 mice were given BLM on day 0, and on Day 14, BM-MSCs were injected into the tail vein. Mice were sacrificed on day 21, and lung tissues were obtained for analysis (a–e). Lung sections were pathologically examined using H&E staining (a) and Masson trichrome staining (b) (section thickness = 3– *μ*m; scale bar = 100 μm, ×100). (c) Collagen content was estimated by hydroxyproline assay. The mRNA expression of collagen I (d) and collagen III (e) was measured by real-time PCR. BM-MSCs(1) = 1 × 10^5^ cells, BM-MSCs(2) = 3 × 10^5^ cells, and BM-MSCs(3) = 1 × 10^6^ cells. G-CSF>BM-MSCs represented BM-MSCs pretreated with G-CSF (30 ng/mL). *n* = 3–5, ^∗^*P* < 0.05, ^∗∗^*P* < 0.01. Bars: mean ± SD.

C57BL/6 mice were given BLM (3.5 mg/kg) on day 0 by intratracheal injection, and on day 14, BM-MSCs with or without G-CSF pretreatment (100 *μ*L; 1 × 10^6^, 3 × 10^6^, or 1 × 10^7^ cells/mL) were injected into the tail vein. Mice were sacrificed on day 21, and lung tissues were obtained for analysis. Severe fibrosis was observed in the BLM group based on H&E and Masson staining, the mRNA expression of collagens I and III, and the HYP content (Figures 1(a)–1(e)). The antifibrotic effect of BM-MSCs (1 × 10^6^, 3 × 10^6^, or 1 × 10^7^ cells/mL) was increased in a dose-dependent manner. The degree of pulmonary fibrosis was significantly reduced in the 3 × 10^6^/mL and 1 × 10^7^/mL BM-MSC groups compared with the BLM group (*P* < 0.05, Figures 1(a)–1(e)), but no antifibrotic effect was observed in the 1 × 10^6^ cells/mL BM-MSC groups. Furthermore, the degree of pulmonary fibrosis in G-CSF-pretreated BM-MSC groups (3 × 10^6^ and 1 × 10^7^ cells/mL) was lower than untreated BM-MSC groups (3 × 10^6^ and 1 × 10^7^ cells/mL) (*P* < 0.05, Figures 1(a)–1(e)). These results indicate that pretreatment with G-CSF could enhance the antifibrotic effect of BM-MSCs.

### 3.2. Pretreatment with G-CSF Promotes the Migration of BM-MSCs to Injured Lung Tissue

**Figure 2:**
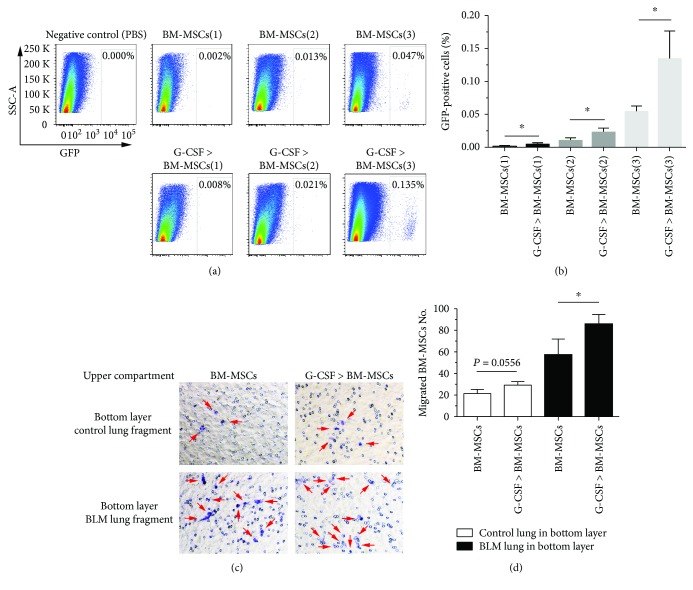
Pretreatment with G-CSF promotes the migration of BM-MSCs to injured lung tissue. Mice received a tail vein injection of GFP-labelled BM-MSCs on day 14, and a single-cell suspension was generated from lung tissue for FCM analysis on day 21 (a, b). (a) Representative FCM analysis of GFP-labelled BM-MSC population in lung tissue. (b) Data analysis of GFP-labelled BM-MSCs population tested by FCM. In the transwell experiment, after allowing the BM-MSCs to migrate for 18 hours, the number of BM-MSCs was calculated under the microscope (c, d). (c) Representative images of migrated BM-MSCs (crystal violet staining: purple). (d) Data analysis of migrated BM-MSCs which are marked with arrows. BM-MSCs(1) = 1 × 10^5^ cells, BM-MSCs(2) = 3 × 10^5^ cells, and BM-MSCs(3) = 1 × 10^6^ cells. G-CSF>BM-MSCs represented BM-MSCs pretreated with G-CSF (30 ng/mL). *n* = 3–5, ^∗^ *P* < 0.05. Bars: mean ± SD.

To explore why G-CSF pretreatment could enhance the antifibrotic effect of BM-MSCs, we observed the effects of transplanting G-CSF-pretreated BM-MSCs on the number of BM-MSCs in BLM-induced pulmonary fibrosis in mice. C57BL/6 mice were given BLM (3.5 mg/kg) on day 0 by intratracheal injection, and on day 14, GFP-labelled BM-MSCs were injected into the tail vein. On day 21, a single-cell suspension was generated from the lung tissue for FCM analysis, and the number of GFP-labelled BM-MSCs in the lung tissue was measured. The results showed that the number of GFP-positive cells in the lung tissue was significantly higher in the G-CSF-pretreated BM-MSC group than in the untreated BM-MSC group (*P* < 0.05, Figures 2(a) and 2(b)).

**Figure 3:**
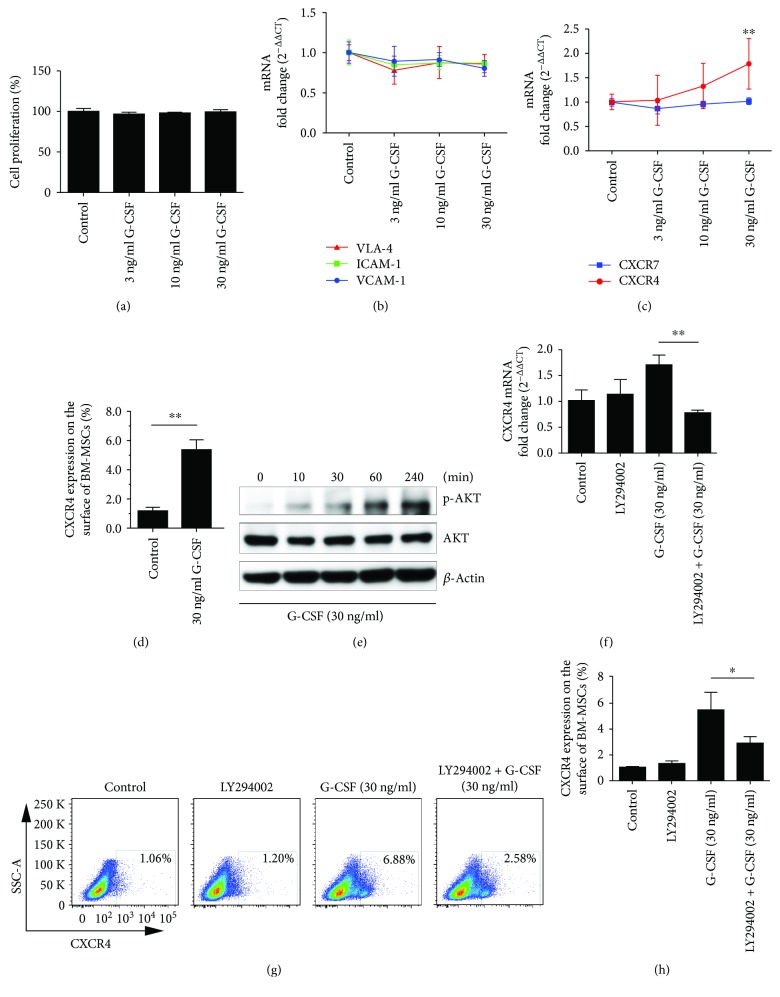
G-CSF promotes CXCR4 expression in BM-MSCs in vitro. The BM-MSCs were treated with G-CSF for 24 h (a–d). (a) The proliferation of BM-MSCs was determined by CCK-8. (b) The mRNA expression of VLA-4, ICAM-1, and VCAM-1 was measured by real-time PCR. (c) The mRNA expression of CXCR4 and CXCR7 was determined by real-time PCR. (d) The population of CXCR4-expressing BM-MSCs was quantified by FCM. (e) Western blot analysis of AKT phosphorylation in BM-MSCs at 0, 10, 30, 60, and 240 min after G-CSF treatment. The BM-MSCs were pretreated with LY294002 for 2 h followed by treatment with G-CSF for 24 h (f–g). (f) The mRNA expression of CXCR4 in BM-MSCs was measured by real-time PCR. (g) Representative FCM analysis of CXCR4-expressing BM-MSC population. (h) Data analysis of CXCR4-expressing BM-MSC population tested by FCM. *n* = 3–6, ^∗^*P* < 0.05, ^∗∗^*P* < 0.01. Bars: mean ± SD.

To determine whether G-CSF could increase the number of BM-MSCs in the lung by promoting the proliferation of BM-MSCs, we initially examined the effect of G-CSF on the proliferation of BM-MSCs in vitro and found no such effect (Figure 3(a)). Next, in order to observe the migration capacity of G-CSF-pretreated BM-MSCs to injured lung tissue, we designed a transwell migration assay. We seeded the BM-MSCs into the upper transwell compartments in 24-well plates. The normal or BLM-induced lung tissue on day 14 after BLM administration was harvested, cut into pieces (1 mm^3^), and incubated into the lower chamber. After that, the cells were allowed to migrate for 18 hours, and the migrated crystal violet-stained BM-MSCs from the series of groups were counted. The quantified data showed that when normal lung tissue was incubated into the lower chamber, G-CSF pretreatment could only slightly increase the migration of BM-MSCs (*P* = 0.0556). However, when BLM-treated lung tissue was incubated into the lower chamber, G-CSF pretreatment significantly increased the migration of BM-MSCs. Furthermore, the chemotaxis ability of BLM-treated lung tissue was significantly higher than that of normal lung tissue (*P* < 0.05; Figures 2(c) and 2(d)). These results suggested that G-CSF could promote the migration and homing of BM-MSCs to injured lung tissue.

### 3.3. G-CSF Promotes the Expression of CXCR4 on BM-MSCs through AKT Signalling Pathway

To further explore the mechanism of which G-CSF promoted BM-MSC migration and homing, we quantified the expression levels of major adhesion molecules (VLA-4, ICAM-1, and VCAM-1) and chemokine receptors (CXCR4 and CXCR7) on BM-MSCs. The results confirmed that G-CSF does not affect the expression of the abovementioned adhesion molecules (Figure 3(b)). The results also showed that CXCR4 expression in BM-MSCs was significantly increased after G-CSF pretreatment, but there was no significant change in CXCR7 expression (Figure 3(c)). Flow cytometry results showed that G-CSF (30 ng/mL) could significantly increase the population of CXCR4-expressing BM-MSCs (*P* < 0.05; Figure 3(d)). Subsequently, we investigated the cell signalling pathways by which G-CSF promotes CXCR4 expression on BM-MSCs. We evaluated AKT signalling pathway activation in BM-MSCs after treatment with G-CSF for different times, and the data indicated that G-CSF could significantly increase AKT phosphorylation (Figure 3(e)). Then, we pretreated BM-MSCs with LY294002, an AKT pathway inhibitor, and observed the effect on G-CSF-regulated CXCR4 expression; the results showed that LY294002 significantly inhibited the upregulation of CXCR4 by G-CSF (*P* < 0.05; Figures 3(f)–3(h)). The results suggested that G-CSF could promote CXCR4 expression by activating the PI3K/AKT signalling pathway.

### 3.4. Knockdown of CXCR4 Expression Reduces the Effect of G-CSF Pretreatment on BM-MSC Migration to Injured Lung Tissue

**Figure 4:**
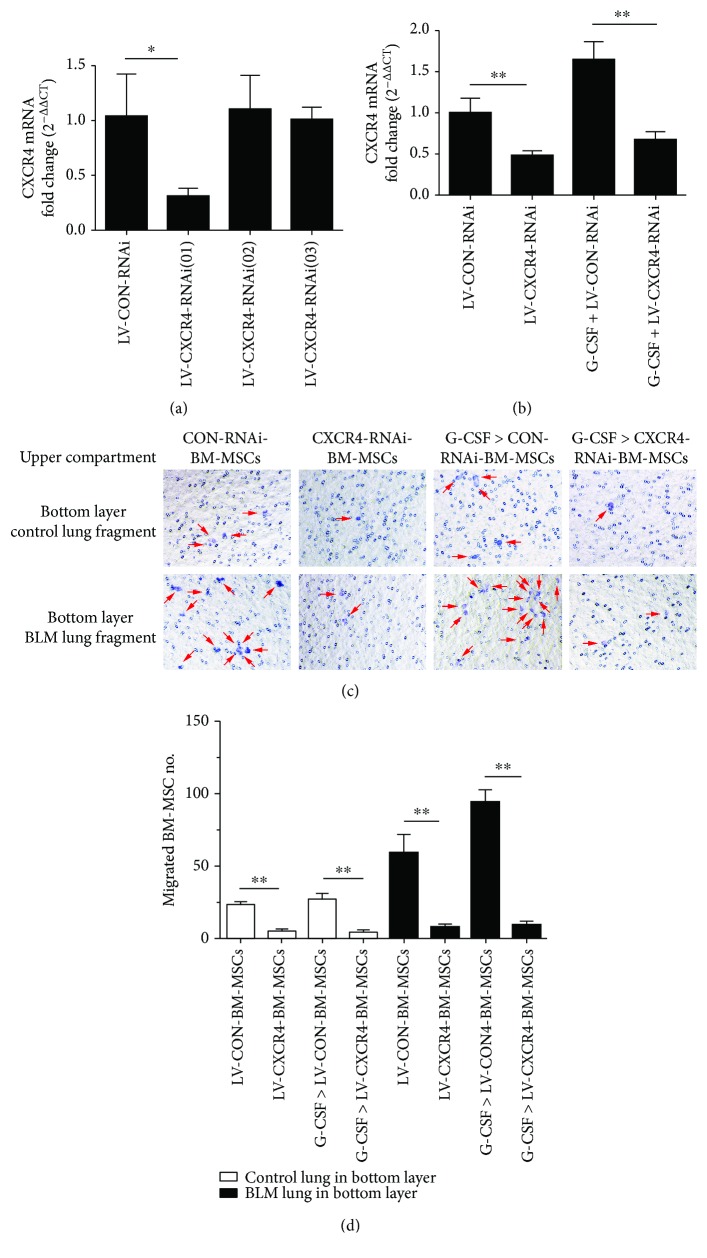
Knockdown of CXCR4 expression reduces the effect of G-CSF-pretreated BM-MSCs on migrating to injured lung tissue. (a) Screening test of CXCR4-RNAi targets. After stably transfecting BM-MSCs with CXCR4-RNAi for 120 h, the mRNA expression of CXCR4 was quantified by real-time PCR. (b) The BM-MSCs transfected with control RNAi or CXCR4-RNAi were treated with or without G-CSF for 24 h; the mRNA expression of CXCR4 was determined by real-time PCR. In the transwell experiment, after allowing the BM-MSCs to migrate for 18 hours, the number of BM-MSCs was calculated under the microscope (c, d). (c) The representative images of migrated BM-MSCs (crystal violet staining: purple). (d) Data analysis of migrated BM-MSCs which are marked with arrows. G-CSF>BM-MSCs represented BM-MSCs pretreated with G-CSF (30 ng/mL). *n* = 3, ^∗^*P* < 0.05, ^∗∗^*P* < 0.01. Bars: mean ± SD.

To further determine the role of the CXCR4 in BM-MSC migration to injured lung tissue, we knocked down CXCR4 in BM-MSCs by transfecting with lentivirus. We first verified the efficacy of the three target sequences. The results of real-time quantitative PCR showed that LV-CXCR4-RNAi (01) significantly reduced CXCR4 mRNA expression levels in BM-MSCs (*P* < 0.05; Figure 4(a)). Then, we treated lentivirus-transfected BM-MSCs with G-CSF (30 ng/mL) for 24 hours and found that CXCR4 mRNA expression was upregulated in the G-CSF-pretreated CON-RNAi-BM-MSC group, which exhibited significantly higher CXCR4 expression than the CON-RNAi-BM-MSC group (*P* < 0.05; Figure 4(b)). Transfecting with LV-CXCR4-RNAi could eliminate the effect of G-CSF on the CXCR4 expression in BM-MSCs, and the CXCR4 mRNA expression level in the G-CSF-pretreated CXCR4-RNAi-BM-MSC group was lower than that in the G-CSF-pretreated CON-RNAi-BM-MSC group. Next, in the transwell experiment, transfected BM-MSCs treated or not with G-CSF were incubated into the upper chamber, and lung tissue was cut into pieces and incubated into the lower chamber. After 18 h, we found that G-CSF pretreatment could enhance the migration of BM-MSCs to injured lung tissue, but this enhancement disappeared when the CXCR4 expression was knocked down (*P* < 0.05; Figures 4(c) and 4(d)). Hence, our results indicated that pretreatment of BM-MSCs with G-CSF promotes the migration of BM-MSCs to the lungs via upregulating the CXCR4 expression.

### 3.5. Knockdown of CXCR4 Expression Reduces the Ability of G-CSF-Pretreated BM-MSCs to Inhibit Pulmonary Fibrosis

**Figure 5:**
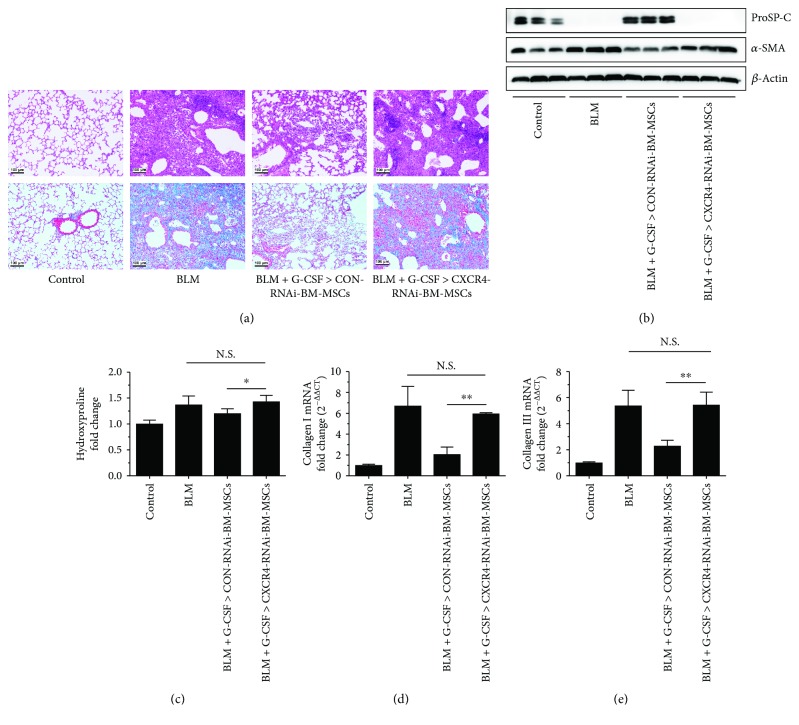
Knockdown of CXCR4 expression reduces the effect of G-CSF-pretreated BM-MSCs on inhibiting pulmonary fibrosis. C57BL/6 mice were given BLM on day 0, and BM-MSCs (1 × 10^6^ cells) were injected into the tail vein on day 14. Mice were sacrificed on day 21, and lung tissues were obtained for analysis (a–e). (a) Pathological lung sections were examined using H&E staining (upper row) and Masson trichrome staining (lower row) (section thickness = 3–4 *μ*m; scale bar = 100 *μ*m, ×100). (b) The protein levels of *α*-SMA and ProSP-C in lung tissue were determined by Western blot. (c) Collagen content was estimated by hydroxyproline assay. The mRNA expression of collagen I (d) and collagen III (e) was determined by real-time PCR. G-CSF>BM-MSCs represented BM-MSCs pretreated with G-CSF (30 ng/mL). *n* = 3–6, ^∗^*P* < 0.05, ^∗∗^*P* < 0.01. Bars: mean ± SD.

C57BL/6 mice were given BLM (3.5 mg/kg) on day 0 by intratracheal injection, and 100 *μ*L of G-CSF-pretreated CXCR4-RNAi-BM-MSCs (1 × 10^7^/mL) or CON-RNAi-BM-MSCs (1 × 10^7^/mL) was injected into the tail vein on day 14, and lung tissues were harvested on day 21. The mouse death rates in the BLM group and G-CSF-pretreated CXCR4-RNAi-BM-MSC transplant group were both up to 40%; however, no deaths were observed in both the control group and G-CSF-pretreated CON-RNAi-BM-MSC transplant group. The H&E and Masson staining, the mRNA expression of collagens I and III, the HYP content, and the protein levels of a-SMA and ProSP-C revealed severe fibrosis in the BLM group compared with the control group (*P* < 0.05; Figures 5(a)–5(e)). However, the degree of lung fibrosis was significantly alleviated in the G-CSF-pretreated CON-RNAi-BM-MSC (1 × 10^7^/mL) group compared with the BLM group and the G-CSF-pretreated CXCR4-RNAi-BM-MSCs (*P* < 0.05; Figures 5(a)–5(e)), but there was no antifibrotic effect when the BLM-induced mice received a tail vein injection of the G-CSF-pretreated CXCR4-RNAi-BM-MSCs (Figures 5(a)-5(e)). These results showed that knocking down the CXCR4 expression in BM-MSCs significantly eliminated the ability of G-CSF to enhance the antifibrotic effects of BM-MSCs.

## 4. Discussion

IPF is a chronic, progressive, fibrotic interstitial pulmonary disease. BLM-induced pulmonary fibrosis in mice is currently the most widely used animal model of pulmonary fibrosis. On days 1–10 after the intratracheal injection of BLM, the mice first develop severe pulmonary inflammation, followed by a gradual decrease in inflammation and an increase in the degree of fibrosis beginning on day 14. Therefore, while most anti-inflammatory drugs can reduce BLM-induced pulmonary fibrosis, they have no significant effect on human IPF. BM-MSCs have been shown to reduce acute pulmonary injury in mice [20]. To exclude the anti-inflammatory effects of BM-MSCs, we transplanted the exogenous BM-MSCs into the mice on day 14 after BLM injection to identify the direct antifibrotic effects of BM-MSCs. The results showed that the transplantation of BM-MSCs effectively reduced BLM-induced pulmonary fibrosis and that G-CSF pretreatment significantly enhanced the antifibrotic effects of BM-MSCs.

G-CSF is one of the most widely used BM stem cell agonists in clinical practice [21]. Studies have shown that G-CSF can promote the repair of many tissues and organs by mobilizing BM stem cells. For example, G-CSF promotes recovery from spinal cord injury in rats by mobilizing BM cells [22]; G-CSF induces BM-HSC mobilization to treat brain injury [23], and the mobilization of BM-MSCs improves damaged myocardium and the treatment of Alzheimer's disease [12, 24]. G-CSF also has a wide range of biological effects and plays important roles in the migration and proliferation of various cells in vitro. For example, G-CSF promotes the migration and angiogenic potential of HUVECs and promotes the proliferation, migration, and invasion of glioma cells [25, 26]. In this study, we transplanted the G-CSF pretreated BM-MSCs into mice with pulmonary fibrosis and observed a significant reduction in the degree of pulmonary fibrosis. We also found that the number of GFP-positive BM-MSCs in the lung tissue in the G-CSF-pretreated group was significantly higher than that in the untreated group. Therefore, we first observed whether G-CSF affects the proliferation, migration, or adhesion ability of BM-MSCs, resulting in a significant increase in BM-MSCs in the lung.

Pitchford et al. showed that G-CSF can significantly promote the in vitro proliferation of BM-HSCs but has little effect on the proliferation of BM-EPCs, indicating that G-CSF has different biological effects on different BM stem cells [27]. There are two possible mechanisms that G-CSF pretreatment increased the number of BM-MSCs in the lung: (1) G-CSF may promote the proliferation of BM-MSCs and (2) G-CSF may promote the migration and homing of BM-MSCs to the lungs. In this study, we initially examined the effect of G-CSF on the proliferation of BM-MSCs and found no such effect. The migration and homing of BM-MSCs to injured tissues are closely related to the expression levels of cell surface adhesion molecules and chemokine receptors [28, 29]. Subsequently, we examined the effects of G-CSF on the expression of major adhesion molecules and chemokine receptors in BM-MSCs. Adhesion molecules can induce circulating stem cells to specifically recognize the matrix microenvironment and homing to the damaged tissue. Chemokines are small cytokines that control the directional movement of cells, and their function is mediated by specific receptors. Therefore, we first examined the expression of the major adhesion molecules VLA-4, ICAM-1, and VCAM-1 in BM-MSCs treated with G-CSF. The results suggested that G-CSF does not affect the expression of the abovementioned adhesion molecules. Next, we further concern about the changes of chemokine receptor expression. SDF-1 is a key chemokine in organisms. Several studies found that the expression of SDF-1 in BLM-induced lungs was significantly increased compared with that in saline-treated lungs [30–33]. CXCR4 is the main receptor for SDF-1 and is expressed on the surface of various stem cells. Recent studies have found that SDF-1 can also bind to CXCR7 [34]. Subsequently, we examined the expression of CXCR4 and CXCR7 in G-CSF-treated BM-MSCs. The results showed that CXCR4 expression on the surface of BM-MSCs was significantly increased after G-CSF pretreatment, but there was no significant change in CXCR7 expression. Therefore, we believe that G-CSF may increase the CXCR4 expression on the surface of BM-MSCs to promote their migration and homing to injured tissues and thereby increase the number of BM-MSCs in damaged lung tissue.

CXCR4 plays an important role in stem cell migration [35]. A previous study found that AMD3100, a specific CXCR4 antagonist, can inhibit the migration of endogenous neural stem cells in rats [36]. Pretreatment with AMD3100 also significantly inhibited the migration of BM-MSCs into injured tissues, thereby limiting the repair ability of BM-MSCs [37, 38]. In this study, we measured the knockdown of CXCR4 to confirm whether CXCR4 mediates the induction of BM-MSC migration into injured pulmonary tissue by G-CSF. The results showed that after knocking down the CXCR4, the ability of G-CSF to promote BM-MSC migration to injured tissues and the inhibitory effect of BM-MSCs on pulmonary fibrosis were significantly reduced, further suggesting that G-CSF promotes BM-MSC activity by upregulating the CXCR4 expression. Migration and homing to injured lung tissue increased the local number of BM-MSCs and enhanced the repair function of these cells. However, the signalling pathway that participates in the regulation of CXCR4 expression by G-CSF has not yet been identified.

The PI3K family contains intracellular phosphatidylinositol kinases that have Ser/Thr kinase activity and are widely expressed in many tissues. Activated PI3K produces a second messenger 3,4,5-triphosphate phosphatidylinositol (PIP3) that triggers a series of phosphorylation reactions that activate AKT. The phosphorylation of AKT activates or inhibits a series of downstream substrates, such as Bad, caspase 9, NF-*κ*B, and GSK23, thereby regulating cell proliferation, differentiation, apoptosis, and migration [39–41]. Furmento et al. found that G-CSF increased MMP-2 and VEGF expression in Swan 71 cells through the activation of PI3K/AKT and thus participated in the regulation of trophoblast function [42]. In this study, G-CSF specifically activated the AKT signalling pathway in BM-MSCs, while pretreatment with the PI3K/AKT inhibitor LY294002 alleviated G-CSF-induced CXCR4 expression. Therefore, we believe that G-CSF promotes the migration and homing of BM-MSCs to injured tissues by promoting CXCR4 expression via the activation of the PI3K/AKT signalling pathway.

Previous studies have suggested that the differentiation of BM-MSCs and their replacement functions are the main factors in promoting the repair of damaged tissue [43]. In BLM-induced lung injury in rats, BM-MSCs can differentiate into alveolar epithelial cells, replace damaged and apoptotic cells, promote tissue repair, and thereby improve pulmonary fibrosis [44]. Recently, researchers have proposed a new hypothesis that BM-MSCs are involved in tissue injury repair in at least two different ways: paracrine mechanisms or differentiation and replacement mechanisms, and the former may play a more crucial role in tissue repair than the latter [45]. Our previous study found that BM-MSCs can inhibit the proliferation and transdifferentiation of fibroblasts by paracrine mechanism, which confirmed that BM-MSCs can alleviate pulmonary fibrosis through a paracrine mechanism [46]. However, no matter what kind of mode of action, BM-MSCs must first migrate and home to the lung. Therefore, our research may provide new ideas for the clinical application of G-CSF and MSCs.

## 5. Conclusions

In summary, our study confirms that G-CSF can promote the migration and homing of BM-MSCs by upregulating the CXCR4 expression, thereby enhancing the antifibrotic effects of BM-MSCs. Thus, our findings provide a new avenue for the application of BM-MSC transplantation in the repair of different tissues in the body.

## Figures and Tables

**Table 1:** The primer sequences for real-time PCR (forward and reverse).

Gene	Forward primer	Reverse primer
*β*-Actin	GGCTGTATTCCCCTCCAT	CCAGTTGGTAACAATGCCATGT
Collagen I	GAGCGGAGAGTACTGGATCG	GCTTCTTTTCCTTGGGGTTC
Collagen III	GCTCCTCTTAGGGGCCACT	CCACGTCTCACCATTGGGG
CXCR4	GGAAACTGCTGGCTGAAAAG	CTGTCATCCCCCTGACTGAT
CXCR7	GCCATGTAACAGCAGCGACT	ATGCCGATCACGAAGATGAA
VCAM-1	CCCAAACAGAGGCAGAGTGT	TGAGCAGGTCAGGTTCACAG
VLA-4	TGTCTGTGTCCCTGTTTGGA	TTTGAGGGGCCTACAGAGAA
ICAM-1	AGATCACATTCACGGTGCTG	CTGGCCTCGGAGACATTAGA

## Data Availability

The data used to support the findings of this study are included within the article.

## Abbreviations

BLM: Bleomycin
IPF: Idiopathic pulmonary fibrosis
G-CSF: Granulocyte colony-stimulating factor
BM-MSCs: Bone marrow mesenchymal stem cells
SDF-1: Stromal cell-derived factor-1
CXCR4: CXC chemokine receptor-4
CXCR7: CXC chemokine receptor-7
TGF-*β*: Transforming growth factor-*β*
HSCs: Hemopoietic stem cells
*α*-SMA: *α*-Smooth muscle actin
VLA-4: Very late antigen-4
VCAM-1: Vascular cell adhesion molecule-1
ICAM-1: Intercellular cell adhesion molecule-1
ProSP-C: Prosurfactant protein C.

## References

[1] Richeldi L., Collard H. R., Jones M. G. (2017). Idiopathic pulmonary fibrosis. *The Lancet*.

[2] Cottin V. (2012). Changing the idiopathic pulmonary fibrosis treatment approach and improving patient outcomes. *European Respiratory Review*.

[3] Weissman I. L., Anderson D. J., Gage F. (2001). Stem and progenitor cells: origins, phenotypes, lineage commitments, and transdifferentiations. *Annual Review of Cell and Developmental Biology*.

[4] Poulsom R., Alison M. R., Forbes S. J., Wright N. A. (2002). Adult stem cell plasticity. *The Journal of Pathology*.

[5] Spees J. L., Lee R. H., Gregory C. A. (2016). Mechanisms of mesenchymal stem/stromal cell function. *Stem Cell Research & Therapy*.

[6] Geiger S., Hirsch D., Hermann F. G. (2017). Cell therapy for lung disease. *European Respiratory Review*.

[7] Hsuan Y. C.-Y., Lin C.-H., Chang C.-P., Lin M.-T. (2016). Mesenchymal stem cell-based treatments for stroke, neural trauma, and heat stroke. *Brain and Behavior: A Cognitive Neuroscience Perspective*.

[8] Gu W., Hong X., Potter C., Qu A., Xu Q. (2017). Mesenchymal stem cells and vascular regeneration. *Microcirculation*.

[9] Eom Y. W., Kim G., Baik S. K. (2015). Mesenchymal stem cell therapy for cirrhosis: present and future perspectives. *World Journal of Gastroenterology*.

[10] Kocher A. A., Schuster M. D., Szabolcs M. J. (2001). Neovascularization of ischemic myocardium by human bone-marrow–derived angioblasts prevents cardiomyocyte apoptosis, reduces remodeling and improves cardiac function. *Nature Medicine*.

[11] Dekker A., Bulley S., Beyene J., Dupuis L. L., Doyle J. J., Sung L. (2006). Meta-analysis of randomized controlled trials of prophylactic granulocyte colony-stimulating factor and granulocyte-macrophage colony-stimulating factor after autologous and allogeneic stem cell transplantation. *Journal of Clinical Oncology*.

[12] Wu C.-C., Wang I.-F., Chiang P.-M., Wang L.-C., Shen C.-K. J., Tsai K.-J. (2017). G-CSF-mobilized bone marrow mesenchymal stem cells replenish neural lineages in Alzheimer’s disease mice via CXCR4/SDF-1 chemotaxis. *Molecular Neurobiology*.

[13] Yannaki E., Athanasiou E., Xagorari A. (2005). G-CSF-primed hematopoietic stem cells or G-CSF per se accelerate recovery and improve survival after liver injury, predominantly by promoting endogenous repair programs. *Experimental Hematology*.

[14] Zhang F., Zhang L., Jiang H. S. (2011). Mobilization of bone marrow cells by CSF3 protects mice from bleomycin-induced lung injury. *Respiration*.

[15] Adachi K., Suzuki M., Sugimoto T. (2002). Granulocyte colony-stimulating factor exacerbates the acute lung injury and pulmonary fibrosis induced by intratracheal administration of bleomycin in rats. *Experimental and Toxicologic Pathology*.

[16] Theus M. H., Wei L., Cui L. (2008). In vitro hypoxic preconditioning of embryonic stem cells as a strategy of promoting cell survival and functional benefits after transplantation into the ischemic rat brain. *Experimental Neurology*.

[17] Chacko S. M., Ahmed S., Selvendiran K., Kuppusamy M. L., Khan M., Kuppusamy P. (2010). Hypoxic preconditioning induces the expression of prosurvival and proangiogenic markers in mesenchymal stem cells. *American Journal of Physiology. Cell Physiology*.

[18] Lan Y. W., Choo K. B., Chen C. M. (2015). Hypoxia-preconditioned mesenchymal stem cells attenuate bleomycin-induced pulmonary fibrosis. *Stem Cell Research & Therapy*.

[19] Zhang W., Liu L., Huo Y., Yang Y., Wang Y. (2014). Hypoxia-pretreated human MSCs attenuate acute kidney injury through enhanced angiogenic and antioxidative capacities. *BioMed Research International*.

[20] Zheng Y., Cai W., Zhou S., Xu L., Jiang C. (2016). Protective effect of bone marrow derived mesenchymal stem cells in lipopolysaccharide-induced acute lung injury mediated by claudin-4 in a rat model. *American Journal of Translational Research*.

[21] Szumilas P., Barcew K., Baskiewicz-Masiuk M., Wiszniewska B., Ratajczak M. Z., Machaliński B. (2005). Effect of stem cell mobilization with cyclophosphamide plus granulocyte colony-stimulating factor on morphology of haematopoietic organs in mice. *Cell Proliferation*.

[22] Urdzíková L., Jendelová P., Glogarová K., Burian M., Hájek M., Syková E. (2006). Transplantation of bone marrow stem cells as well as mobilization by granulocyte-colony stimulating factor promotes recovery after spinal cord injury in rats. *Journal of Neurotrauma*.

[23] Mehrdad B., Mohsen M., Mehdi M. (2010). Comparison of transplantation of bone marrow stromal cells (BMSC) and stem cell mobilization by granulocyte colony stimulating factor after traumatic brain injury in rat. *Iranian Biomedical Journal*.

[24] Kawada H., Fujita J., Kinjo K. (2004). Nonhematopoietic mesenchymal stem cells can be mobilized and differentiate into cardiomyocytes after myocardial infarction. *Blood*.

[25] Huang H., Zhang Q., Liu J., Hao H., Jiang C., Han W. (2017). Granulocyte-colony stimulating factor (G-CSF) accelerates wound healing in hemorrhagic shock rats by enhancing angiogenesis and attenuating apoptosis. *Medical Science Monitor*.

[26] Wang J., Yao L., Zhao S. (2012). Granulocyte-colony stimulating factor promotes proliferation, migration and invasion in glioma cells. *Cancer Biology & Therapy*.

[27] Pitchford S. C., Furze R. C., Jones C. P., Wengner A. M., Rankin S. M. (2009). Differential mobilization of subsets of progenitor cells from the bone marrow. *Cell Stem Cell*.

[28] Bolno P. B., Morgan D., Wechsler A., Kresh J. Y. (2004). Chemokine induced migration of human mesenchymal stem cells: a strategy for directing cardiac repair. *Journal of the American College of Surgeons*.

[29] Cecyn K. Z., Kimura E. Y. S., Lima D. M. S. M., Yamamoto M., Bordin J. O., de Oliveira J. S. R. (2018). Expression of adhesion molecules on CD34+ cells from steady-state bone marrow before and after mobilization and their association with the yield of CD34+ cells. *Blood Research*.

[30] Hashimoto N., Jin H., Liu T., Chensue S. W., Phan S. H. (2004). Bone marrow–derived progenitor cells in pulmonary fibrosis. *Journal of Clinical Investigation*.

[31] Song J. S., Kang C. M., Kang H. H. (2010). Inhibitory effect of CXC chemokine receptor 4 antagonist AMD3100 on bleomycin induced murine pulmonary fibrosis. *Experimental & Molecular Medicine*.

[32] Chen Y., Yu X., He Y. (2017). Activation of A2aR attenuates bleomycin-induced pulmonary fibrosis via the SDF-1/CXCR4 axis-related pathway. *American Journal of Translational Research*.

[33] Xu J., Li L., Xiong J., Zheng Y., Ye Q., Li Y. (2015). Cyclophosphamide combined with bone marrow mesenchymal stromal cells protects against bleomycin-induced lung fibrosis in mice. *Annals of Clinical and Laboratory Science*.

[34] Burns J. M., Summers B. C., Wang Y. (2006). A novel chemokine receptor for SDF-1 and I-TAC involved in cell survival, cell adhesion, and tumor development. *The Journal of Experimental Medicine*.

[35] Takeuchi H., Natsume A., Wakabayashi T. (2007). Intravenously transplanted human neural stem cells migrate to the injured spinal cord in adult mice in an SDF-1- and HGF-dependent manner. *Neuroscience Letters*.

[36] Liu J. M., Zhao K., Du L. X. (2017). AMD3100 inhibits the migration and differentiation of neural stem cells after spinal cord injury. *Scientific Reports*.

[37] Liu N., Patzak A., Zhang J. (2013). CXCR4-overexpressing bone marrow-derived mesenchymal stem cells improve repair of acute kidney injury. *American Journal of Physiology Renal Physiology*.

[38] Hu C., Yong X., Li C. (2013). CXCL12/CXCR4 axis promotes mesenchymal stem cell mobilization to burn wounds and contributes to wound repair. *The Journal of Surgical Research*.

[39] Yu J., Li M., Qu Z., Yan D., Li D., Ruan Q. (2010). SDF-1/CXCR4-mediated migration of transplanted bone marrow stromal cells toward areas of heart myocardial infarction through activation of PI3K/Akt. *Journal of Cardiovascular Pharmacology*.

[40] Zheng H., Fu G., Dai T., Huang H. (2007). Migration of endothelial progenitor cells mediated by stromal cell-derived factor-1α/CXCR4 via PI3K/Akt/eNOS signal transduction pathway. *Journal of Cardiovascular Pharmacology*.

[41] Wang H., Yin Y., Li W. (2012). Over-expression of PDGFR-β promotes PDGF-induced proliferation, migration, and angiogenesis of EPCs through PI3K/Akt signaling pathway. *PLoS One*.

[42] Furmento V. A., Marino J., Blank V. C., Roguin L. P. (2014). The granulocyte colony-stimulating factor (G-CSF) upregulates metalloproteinase-2 and VEGF through PI3K/Akt and Erk1/2 activation in human trophoblast Swan 71 cells. *Placenta*.

[43] Wu Y., Wang J. F., Scott P. G., Tredget E. E. (2007). Bone marrow-derived stem cells in wound healing: a review. *Wound Repair and Regeneration*.

[44] Zhao F., Zhang Y. F., Liu Y. G. (2008). Therapeutic effects of bone marrow-derived mesenchymal stem cells engraftment on bleomycin-induced lung injury in rats. *Transplantation Proceedings*.

[45] Fox J. M., Chamberlain G., Ashton B. A., Middleton J. (2007). Recent advances into the understanding of mesenchymal stem cell trafficking. *British Journal of Haematology*.

[46] Shen Q., Chen B., Xiao Z. (2015). Paracrine factors from mesenchymal stem cells attenuate epithelial injury and lung fibrosis. *Molecular Medicine Reports*.

